# Clinical Infections by Herpesviruses in Patients Treated with Valproic Acid: A Nested Case-Control Study in the Spanish Primary Care Database, BIFAP

**DOI:** 10.3390/jcm8091442

**Published:** 2019-09-11

**Authors:** Miguel Gil, Rocío González-González, Angela Vázquez-Calvo, Arturo Álvarez-Gutiérrez, Miguel A. Martín-Acebes, Beatriz Praena, Raquel Bello-Morales, Juan-Carlos Saiz, Jose A. López-Guerrero, Enrique Tabarés, Francisco Sobrino

**Affiliations:** 1 BIFAP, Division of Pharmacoepidemiology and Pharmacovigilance, Spanish Agency for Medicines and Medical Devices (AEMPS), 28022 Madrid, Spain; rogogo@gmail.com (R.G.-G.); aalvarez_externo@aemps.es (A.Á.-G.); 2 Centro de Biología Molecular Severo Ochoa (CSIC-UAM), Nicolás Cabrera 1, 28049 Madrid, Spain; angelavazquezcalvo@gmail.com (A.V.-C.); bpraena@cbm.csic.es (B.P.); raquel.bello-morales@inv.uam.es (R.B.-M.); jal@cbm.uam.es (J.A.L.-G.); 3 Department of Biotechnology, Instituto Nacional de Investigación y Tecnología Agraria y Alimentaria (INA), 28040 Madrid, Spain; martin.mangel@inia.es (M.A.M.-A.); jcsaiz@inia.es (J.-C.S.); 4 Universidad Autónoma de Madrid, Departamento de Biología Molecular, Edificio de. Biología, Darwin 2, Cantoblanco, 28049 Madrid, Spain; 5 Universidad Autónoma de Madrid, Facultad de Medicina, Arzobispo Morcillo 4, 28029 Madrid, Spain; enrique.tabares@uam.es

**Keywords:** BIFAP, valproic acid, herpesvirus infections, case-control study, electronic health care database

## Abstract

The objective of this study is to evaluate the risk of clinical infections by herpesviruses in patients exposed to valproic acid (VPA). We performed a case-control study nested in a primary cohort selected from the Spanish primary care population-based research database BIFAP (Base de datos para la Investigación Farmacoepidemiológica en Atención Primaria) over the period 2001–2015. The events of interest were those diseases caused by any herpesviruses known to infect humans. For each case, up to 10 controls per case matched by age, gender, and calendar date were randomly selected. A conditional logistic regression was used to compute adjusted odds ratios (OR) and their 95% confidence intervals (95% CI). Current use of VPA was associated with a trend towards a reduced risk of clinical infections by herpesviruses as compared with non-users (OR 0.84; CI 95% 0.7–1.0; *p* = 0.057). Among current users, a trend to a decreased risk with treatment durations longer than 90 days was also observed. The results show a trend to a reduced risk of clinical infection by herpesviruses in patients exposed to VPA. These results are consistent with those in vitro studies showing that, in cultured cells, VPA can inhibit the production of the infectious progeny of herpesviruses. This study also shows the efficient use of electronic healthcare records for clinical exploratory research studies.

## 1. Introduction

Information derived from databases containing electronic health records (EHR) is increasingly being used to conduct pharmacoepidemiologic research. EHRs’ usage is broad, including safety surveillance, comparative effectiveness, drug utilization, and so on [1]. A potential and less developed use of EHRs is the study of new indications of drugs already marketed, either to translate findings in basic research into medical practice (translational research) or to support findings in other clinical studies.

Short chain fatty acid valproic acid (VPA) is an active substance commonly used in epilepsy and other psychiatric and neurological disorders, including bipolar disorder, neuropathic pain, and migraine [2]. VPA and related substances with licensed products in Spain are valproate sodium and valpromide. In vitro studies have shown that VPA inhibits the production of infectious progeny of a broad spectrum of enveloped viruses causing human and veterinary diseases, which likely reflects a VPA-mediated impairment of lipid metabolism that may affect different steps of a virus life cycle [3,4,5,6,7,8,9].

Enveloped viruses include a broad range of virus families. Among them, Herpesviridae is a large family of DNA viruses including different species that cause diseases in humans with a high burden of disease, such as herpes simplex virus (HHV-1 and HHV-2) causing orolabial and genital herpes, varicella zoster virus causing varicella, or diseases caused by infections by Epstein–Barr virus (mononucleosis/some cancers) and cytomegalovirus.

VPA doses usually administered to humans are dependent on the body mass, with a therapeutic range for epilepsy treatment of 50 to 100 mg/liter (about 0.3 to 0.6 mM) in plasma. Some studies have shown the possibility of the therapeutic antiviral use of the VPA based on its half maximal inhibitory concentration (IC50) and selectivity index (SI) [9]. Indeed, an IC50 of 0.55 mM has been determined for VPA inhibition of HHV-1 in human oligodendroglioma (HOG) cultured cells, which is compatible with a potential in vivo antiviral effect of VPA [4].

In spite of in vitro findings, to the best of our knowledge, no clinical studies in humans are available testing the hypothesis of an antiviral effect of the VPA.

The objective of this study is to evaluate the risk of clinical infections by the viruses of the family Herpesviridae (herpesviruses) in patients exposed to VPA in the Spanish research database BIFAP (Base de datos para la Investigación Farmacoepidemiológica en Atención Primaria).

## 2. Experimental Section

### 2.1. Data Source Description

The study was performed in the Spanish primary care population-based research database BIFAP. BIFAP is a non-profit program of the Spanish Agency of medicines and medical devices in collaboration with nine autonomous regions of Spain (www.bifap.org) [10]. The BIFAP research database for the year 2014 includes anonymized data from over 7.6 million patients prospectively and routinely registered in the electronic healthcare records (EHR) by 5714 primary care physicians (PCPs), both general practitioners and pediatricians, over the period 2001–2015. The mean follow-up of patients included in BIFAP database is 5.1 years, totalling 38.6 million person-years of follow-up. BIFAP is comparable to the Spanish population with respect to its age and sex distribution, covering 17.0% of the total Spanish population. BIFAP has been validated for pharmacoepidemiologic research through multiple studies [10] and successfully compared to other well-known European databases [11,12,13,14].

The information recorded in BIFAP includes demographic data, clinical data, drug prescriptions, referrals to specialists, clinical notes as free-text, and other additional health data (i.e., test results, interventions, lifestyle information). Prescriptions are coded according to the Anatomical Therapeutic Classification (ATC), and the following data are recorded: product name, date of prescription, quantity prescribed, dose regime, and duration of drug therapy.

### 2.2. Study Design

We performed a case-control study nested in a primary cohort selected from BIFAP over the period 1 January 2001 to 31 December 2015. The study cohort included those patients aged 100 years or lower, with at least two years of continuous enrolment with the primary care physician. Each patient started his/her follow-up in the study cohort when he/she met those criteria (start date). We were interested in the first ever episodes of the events of interest among new users of VPA in the study period [15]. Thus, patients were excluded from the study cohort if they had, previous to the start date of follow-up, a registry of clinical infections by herpesviruses, varicella vaccination registry, or a prescription of an antiepileptic drug. Likewise, patients with a previous history of cancer were excluded given their characteristics and the likely lack of exhaustive information in primary care records.

The population of the study cohort (*n* = 5,858,722) was then followed-up until the earliest occurrence one of the following: incident disease caused by any of the different herpesviruses assessed (event of interest), 101 years old, cancer diagnosis, death, varicella vaccination registry, end of follow-up of the patient, or end of the study period.

### 2.3. Case Identification

The events of interest were those diseases caused by any of the herpesviruses known to infect humans, specifically the following:

- Orolabial herpes and genital herpes: caused by HHV-1 and 2 (herpes simplex virus);
- Varicella: caused by HHV-3 (varicella-zoster virus);
- Infectious mononucleosis: caused by HHV-4 (Epstein–Barr virus) or HHV-5 (human cytomegalovirus);
- Roseola infantum: caused by HHV-6;
- Kaposi’s sarcoma: caused by HHV-8 (Kaposi’s sarcoma-associated herpesvirus).

Clinical data are coded in BIFAP in accordance with two diagnostic coding systems: the International Classification of Primary Care (ICPC) and the International Classification of Diseases (ICD-9). The ICPC dictionary is used in most of the autonomous communities involved in BIFAP and its granularity is limited (686 codes), as compared with the ICD-9 dictionary (23,222 codes). For ICPC, a more granular dictionary is available for research purposes in BIFAP (ICPC-BIFAP). The detailed description of the procedures to build the ICPC-BIFAP is included in Appendix A.

Case-finding algorithms (CFA) were built for each of the events of interest based on proper code selection in those dictionaries plus text-mining strategies. In Appendix B, the specific ICD-9 codes (Table A1), ICPC-BIFAP codes (Table A2), and string text search criteria (Table A3) are detailed. The first registry of any of the above mentioned events of interest during the follow-up was identified to be used as case in the study, and the recorded date of that event was considered as the index date for the study.

Additional text-mining techniques were performed in the clinical notes linked to the diagnosis, in order to identify non-cases among those events selected by the defined case-finding algorithm (false positives). Text mining included a broad search of semantic terms related to “vaccination”, “previous history”, “relatives”, “exposure/contact”, and so on. Clinical profiles of the cases meeting these criteria were reviewed manually and those confirmed as non-cases were disregarded for the study.

### 2.4. Selection of Controls

Up to 10 controls per case matched by age (+/− 1 year), gender, and calendar date were randomly selected from the risk set for each case (risk set sampling). Accordingly, a subject might be selected as control before being a case and might be selected as a control for more than one case. The date of the herpesvirus infection (case) was considered as the index date for the matched controls.

### 2.5. Exposure Definition

Exposure of interest included VPA and related substances (sodium valproate and valpromide).

We categorized cases and controls as current users when the supply of prescription finished within 30 days before the date of the herpesvirus infection or the corresponding date for the matched controls (index dates); recent users when supply finished between 31 and 365 days before the index date; past users when supply finished more than one year before the index date; and nonusers when there was no recorded prescription ever.

The effect of treatment duration among current users was also evaluated. We considered prescriptions to be consecutive when the time elapsed between the end of supply of one prescription and the start of the next was 90 days or less. Continuous duration was categorized into the following time-windows: <91 days, 91–365 days, and >365 days.

### 2.6. Potential Confounding Variables

Potential confounding variables considered for the analysis included the previous history of the following comorbidities and risk factors any time previous to the index date of cases and matched controls:

- Diseases affecting the immune system; Human Immunodeficiency Virus (HIV) infection, other immunodeficiencies;
- Chronic diseases impairing health conditions: asthma, chronic obstructive pulmonary disease, ischaemic heart disease, stroke, diabetes mellitus, heart failure, hypertension, hyperlipidemia, chronic hepatitis, chronic renal failure, and Alzheimer disease;
- Lifestyle conditions: alcohol abuse, smoking, body mass index, and obesity;
- Diseases related to the potential use of VPA: depression, neuropathic pain, epilepsy, bipolar disorder, and migraine;
- Comedications: systemic corticosteroids, systemic antiviral drugs, immunosuppressant, antibiotics, anxiolytics, antipsychotic drugs, and antiepileptic drugs other than VPA.
- The number of visits to the PCP (<6, 6–15, 16–24, >24) was ascertained in the two-year period before the index date.

### 2.7. Statistical Analysis

Cases and controls were matched by age, sex, and index date. We used conditional logistic regression to compute adjusted odds ratios (OR) and their 95% confidence intervals (95% CI) for infection by herpesviruses associated to the use of VPA after adjusting for the potential confounders described above.

As sensitivity analysis, separate analyses were also performed to assess the association for the different herpesviruses grouped according the following human diseases: orolabial/genital (HHV-1/HHV-2); varicella/herpes zoster (HHV-3); infectious mononucleosis (HHV-4/HHV-5); roseola infantum (HHV-6), and Kaposi’s sarcoma (HHV-8).

In addition, the effect of valpromide, a VPA derivate, was also evaluated separately in order to test if a differential effect was observed.

The level of statistical significance was *p* < 0.05. Statistical analyses were performed using Stata (version 15, StataCorp LLC, College Station, TX, USA).

### 2.8. Ethics Review

The scientific committee of BIFAP granted a positive opinion of the study protocol (#08/2015). The investigators had access to only fully anonymized data and, under this condition, no specific ethics review was required according to Spanish law.

## 3. Results

### 3.1. Characteristics of the Study Cohort, Validation, and Incidence of Clinical Infections by Herpesviruses in BIFAP Database

**Figure 1:**
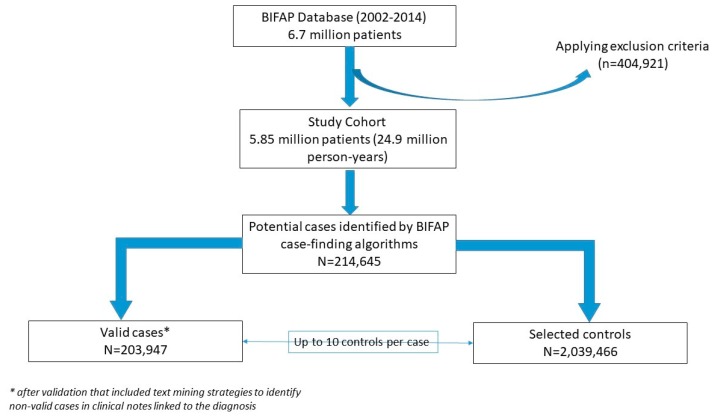
Flow chart showing the identification processes of cases and controls in the Base de datos para la Investigación Farmacoepidemiológica en Atención Primaria (BIFAP) database.

The study cohort was made up of 5,858,722 patients, totalling 24,932,043 person-years (py) of follow-up. With the initial computer search (BIFAP case-finding algorithms), 214,645 potential cases were retrieved. Among them, 10,698 were excluded after additional validation using text mining strategies in the clinical notes linked to the diagnosis. The resulting number of valid cases for the study was of 203,947. The flowchart for the selection of cases is displayed in Figure 1.

Most of the valid cases of clinical infections by herpesviruses included in the study were those produced by HHV-1/HHV-2 or HHV-3 causing orolabial/genital herpes and varicella, respectively, in humans (see Table 1).

The resulting incidence of clinical infections by herpesviruses in the BIFAP database was 8.1 per 1000 person-years of follow-up. This incidence was higher for orolabial/genital herpes (4.1 per 1000 py) and varicella/herpes Zoster (3.5 per 1000 py) infections, respectively.

### 3.2. Characteristics of Cases and Controls Included in the Study

Our study population included 203,947 cases and 2,039,466 controls. Most cases were infections caused by HHV-1/HHV-2 (49.9%) and HHV-3 (42.7%), causing orolabial/genital herpes and varicella/herpes zoster simple, respectively (see Table 1). Among these cases, 58.4% were male and 43.5% were younger than 10 years old (see Table 2). The cases, in general, presented a greater proportion of comorbidities and drug use than controls. The distribution of cases and controls at the index date of demographic characteristics (matching variables), lifestyle/comorbidities, and previous use of medications are shown in Table 2, Table 3 and Table 4, respectively.

### 3.3. Risk of Clinical Infection by Herpesviruses Associated to the Use of VPA

Current use of VPA was associated with a trend to a reduced risk of clinical infection by herpesviruses as compared with non-users (OR 0.84; 95% CI 0.7–1.0, *p* = 0.057). This trend was also observed for those who abandon the medication in the first year (recent use OR 0.79; CI 95% 0.60–1.02, *p* = 0.071). Among current users, a trend to a decreased risk with treatment durations longer than 90 days was observed, being statistically significant for durations longer than one year (OR 0.68; 95% CI 0.48–0.95, *p* = 0.02) (see Table 5).

Concerning the risk of the diverse clinical diseases caused by the different herpesviruses, a non-statistically significant reduced risk in current users of VPA was observed for orolabial/genital herpes (OR 0.89; 95% CI 0.70–1.15; *p* = 0.37) and varicella (OR 0.92; 95% CI 0.68–1.24; *p* = 0.58) (see Table 6). Both diseases represent 92.6% of cases included in the study. Separate evaluation was not feasible for roseola infantum or Kaposi’s sarcoma owing to the low number of exposed cases.

The trend to a decreased risk among current users observed with treatment durations longer than 90 days for all herpesviruses (see Table 5) was only noticed for orolabial/genital herpes (HHV-1/HHV-2) (see Table 6). For infectious mononucleosis (HHV-4/HHV-5), a non-significant decreased risk was observed for treatment durations longer than 365 days (OR 0.15; 95% CI 0.02–1.16; *p* = 0.07) (see Table 6).

Patients currently exposed to valpromide represent a low percentage (2.6%) of those exposed to VPA and related substances. Separate results for valpromide showed also a decreased risk of infections by herpesviruses (OR 0.60; 95% CI, 0.18–2.00), although the low number of cases of controls exposed (3 and 30, respectively) led to wide confidence intervals.

## 4. Discussion

The results of this study show a trend to a reduced risk of clinical infections by herpesviruses in patients exposed to VPA, especially in those with treatment durations longer than 90 days. This trend to a reduced risk is consistent across the most frequent herpesviruses infections, although differences were not statistically significant.

These results are consistent with in vitro studies showing that, in cultured cells, VPA inhibits the production of infectious progeny of different enveloped viruses causing relevant human and veterinary diseases [3,5,6,9,16,17] including herpesviruses [4,7,8].

To the best of our knowledge, this is the first epidemiological study designed to test the in vitro hypothesis of an antiviral effect of VPA. In the context of this translational research, a study was performed resulting in an IC50 of 0.55 mM for VPA inhibition of HSV-1 in HOG cultured cells, which is compatible with a potential antiviral effect in vivo of VPA [4]

Several studies have shown that VPA can stimulate the infectivity and replication of enveloped viruses, including some of the Herpesviridae family studied here [8,18]. Enhancement of herpesvirus infection has been reported in cells competent for Human type I interferons (IFN) induction upon VPA treatment in a process associated with the VPA-mediated inhibition of type I histone deacetylases (HDAC) [19,20,21]. Histone deacetylation is required for expression of IFN-stimulated genes (ISGs) [22]. Consistently, HADC inhibition by VPA results in ISGs’ downregulation and a decrease in type I IFN response [23]. However, the present analysis does not support an increase of the incidence of herpesvirus infections in patients treated with VPA, but is consistent with the proposed inhibitory effect of VPA on the infection of herpesviruses [4].

The results of our epidemiological study are consistent, and extend to humans, with previous reports on the in vivo potential of VPA and its derivative valpromide—which lacks the free carboxylic group and the HADC inhibitory activity—to interfere with herpesvirus multiplication in mice [7]. Hence, a trend to a protective effect in human patients was observed for both compounds, although the decreased risk observed was marginally significant only for VPA (*p* = 0.053). Separate estimations for valpromide were based in a few number of cases and controls exposed, leading to wide confidence intervals.

Interestingly, the trend towards a protective effect observed extends to the most common diseases caused by the different herpesviruses like orolabial/genital herpes and varicella, although differences were not statistically significant because of the limited number of exposed events (see Table 6). Varicella represents 42.7% of cases in the study. The incidence of varicella in BIFAP (4.1 per 1000 py) was comparable to that reported to the Spanish network of epidemiological surveillance [24], suggesting an exhaustive registration of Varicella in the BIFAP database. The Spanish Association of Pediatricians has recommended varicella vaccination for all children since the year 2000. Nevertheless, varicella vaccination was not included in the official vaccination calendar in Spain until the year 2016, being fully financed by the Spanish National Health Service in the year 2018 [25]. Thus, a substantial decrease in the burden of disease is expected for the coming years in Spain.

Regarding the effect of VPA treatment duration, the trend to a decreased risk among current users with treatment durations longer than 90 days observed for all herpesviruses (see Table 5) was only noticed for orolabial/genital herpes and, to a lesser extent, for infectious mononucleosis (see Table 6), although differences were not statistically significant. Thus, VPA might have different functions on multiple types of herpesviruses and their associated diseases, although further study is necessary to confirm this hypothesis.

Most herpesviruses infections are asymptomatic and seroconversion rates in humans are high. Multiple factors might be involved to develop clinical disease among those infected, but asymptomatic patients. Also, VPA itself might, theoretically, have a role in this, although neither previous hypothesis nor potential mechanisms have been reported.

The patient’s seroconversion status is not usually available in clinical EHR and the events caused by herpesviruses evaluated in this study are only those with clinical symptoms. Consequently, the statistical analysis cannot be additionally adjusted/stratified by asymptomatic infection. Nevertheless, given the previous considerations, an imbalance in the proportion of patients with an asymptomatic infection among cases and controls regarding exposure status is not expected.

The combined evidence of in vitro studies, IC50, and epidemiological studies/data supports a potential therapeutic antiviral use of VPA to treat infections caused by herpesviruses. The trend to a protective effect of VPA in human patients found is marginally significant and, given the observational nature of the study, the possibility of residual confounding by unmeasured factors cannot be ruled out. Thus, further research is needed to confirm these findings.

However, any further development or study to evaluate the therapeutic effect of VPA is jeopardized by the risk of neurodevelopmental disorders and congenital malformations in children exposed intra-utero to VPA [26]. Given the great public impact of some of the diseases caused by enveloped virus and the potential protective effect of VPA on the risk of herpesviruses infections in the general population, it might be helpful to understand the mechanism behind these protective effects and to explore the finding of safer related compounds.

In summary, this study demonstrates that well-organized and updated databases from health records could be efficiently used for clinical exploratory studies in order to investigate the potential secondary use of drugs already in clinical practice and design targeted clinical trials

## 5. Conclusions

In conclusion, the current study shows how translational research is helpful in order to strengthen in vitro hypotheses in humans using electronic health care records, and suggest a potential effect of VPA on clinical diseases caused by an enveloped virus of the Herpesviridae family.

## Figures and Tables

**Table 1:** Herpesvirus infections type distribution for cases and controls.

Herpes Virus Type	Disease in Humans	Controls (*N* = 2,039,466)	Cases (*N* = 203,947)
**HHV-1/HHV-2**	Oral/genital herpes	1,018,000 (49.9%)	101,800 (49.9%)
**HHV-3**	Varicella/herpes zoster	869,906 (42.7%)	86,991 (42.7%)
**HHV-4/HHV-5**	Infectious mononucleosis	146,030 (7.2%)	14,603 (7.2%)
**HHV-6**	Roseola infantum	5390 (0.3%)	539 (0.3%)
**HHV-8**	Kaposi’s sarcoma	140 (0.007%)	14 (0.007%)

HHV: Herpesviruses.

**Table 2:** Age and sex distribution (matching variables) of cases and controls.

Sociodemographic Variable	Categories	Cases (*n* (%)) *N* = 203,947	Controls (*n* (%)) *N* = 2,039,466
Age	0 year	122,452 (6%)	9317 (4.57%)
	1–4 years	529,832 (25.98%)	56,789 (27.84%)
	5–9 years	233,747 (11.46%)	22,660 (11.11%)
	10–17 years	135,515 (6.64%)	13,465 (6.6%)
	18–44 years	557,298 (27.33%)	55,627 (27.28%)
	45–64 years	283,505 (13.9%)	28,318 (13.88%)
	65–74 years	103,221 (5.06%)	10,361 (5.08%)
	75+	73,896 (3.62%)	7410 (3.63%)
Gender	Male	1,190,590 (58.38%)	119,059 (58.38%)
	Female	848,876 (41.62%)	84,888 (41.62%)

Cases and controls were matched by age and sex.

**Table 3:** Lifestyle and comorbidities distribution between cases and controls.

Lifestyle/Comorbidities	Categories	Cases (*n* (%)) *N* = 203,947	Controls (*n* (%)) *N* = 2,039,466	Crude OR (95%CI)
Number of visits to the PCP (last 3 years)	1–5	686,728 (33.67%)	26,081 (12.79%)	1 (ref)
	6–15	696,274 (34.14%)	73,880 (36.23%)	3.14 (3.09–3.18)
	16–24	332,186 (16.29%)	46,920 (23.01%)	4.85 (4.77–4.93)
	25+	324,278 (15.9%)	57,066 (27.98%)	6.78 (6.67–6.90)
Smoking	Non smoker	271,264 (13.3%)	30,853 (15.13%)	1 (ref)
	Ex-smoker	27,752 (1.36%)	3673 (1.8%)	1.19 (1.14–1.23)
	Current smoker	194,223 (9.52%)	20,395 (10%)	0.92 (0.90–0.94)
	Missing information	1,546,227 (75.82%)	149,026 (73.07%)	0.81 (0.80–0.83)
Alcohol consumption	No	1,533,808 (75.21%)	147,434 (72.29%)	1 (ref)
	Yes	505,658 (24.79%)	56,513 (27.71%)	1.24 (1.23–1.26)
Chronic obstructive pulmonary disease	No	2,021,282 (99.11%)	201,892 (98.99%)	1 (ref)
	Yes	2055 (1.01%)	18,184 (0.89%)	1.14 (1.09–1.20)
Asthma	No	181,273 (88.88%)	1,874,907 (91.93%)	1 (ref)
	Yes	164,559 (8.07%)	22,674 (11.12%)	1.44 (1.42–1.46)
Ischaemic heart disease	No	2,019,995 (99.05%)	201,768 (98.93%)	1 (ref)
	Yes	19,471 (0.95%)	2179 (1.07%)	1.13 (1.08–1.18)
Cerebrovascular accident	No	2,022,049 (99.15%)	202,073 (99.08%)	1 (ref)
	TIA	12,405 (0.61%)	1233 (0.6%)	1 (0.94–1.06)
	Stroke	5012 (0.25%)	641 (0.31%)	1.29 (1.18–1.40)
Diabetes Mellitus	No	1,973,842 (96.78%)	197,431 (96.81%)	1 (ref)
	Yes	65,624 (3.22%)	6516 (3.19%)	0.99 (0.96–1.02)
Heart failure	No	2,032,388 (99.65%)	203,246 (99.66%)	1 (ref)
	Yes	7078 (0.35%)	701 (0.34%)	0.99 (0.91–1.07)
Hypertension	No	1,856,925 (91.05%)	184,572 (90.5%)	1 (ref)
	Yes	182,541 (8.95%)	19,375 (9.5%)	1.11 (1.09–1.14)
Hyperlipidemia	No	1,845,499 (90.49%)	181,358 (88.92%)	1 (ref)
	Yes	193,967 (9.51%)	22,589 (11.08%)	1.28 (1.25–1.30)
Chronic renal disease	No	2,032,754 (99.67%)	203,196 (99.63%)	1 (ref)
	Yes	6712 (0.33%)	751 (0.37%)	1.12 (1.04–1.21)
AIDS/ HIV+	No	2,037,298 (99.89%)	203,427 (99.75%)	1 (ref)
	Yes	2168 (0.11%)	520 (0.25%)	2.41 (2.19–2.66)
Chronic hepatitis	No	2,034,550 (99.76%)	203,355 (99.71%)	1 (ref)
	Yes	4916 (0.24%)	592 (0.29%)	1.21 (1.11–1.31)
Alzheimer disease	No	2,032,190 (99.64%)	203,350 (99.71%)	1 (ref)
	Yes	7276 (0.36%)	597 (0.29%)	0.81 (0.74–0.88)
Immunodeficiency	No	2,038,212 (99.94%)	203,744 (99.9%)	1 (ref)
	Yes	1254 (0.06%)	203 (0.1%)	1.62 (1.40–1.88)
Depression	No	1,854,589 (90.94%)	179,097 (87.82%)	1 (ref)
	Yes	184,877 (9.06%)	24,850 (12.18%)	1.49 (1.47–1.51)
Neuropathic pain	No	2,030,151 (99.54%)	202,443 (99.26%)	1 (ref)
	Yes	9315 (0.46%)	1504 (0.74%)	1.63 (1.54–1.72)
Epilepsy	No	2,025,726 (99.33%)	202,001 (99.05%)	1 (ref)
	Yes	13,740 (0.67%)	1946 (0.95%)	1.43 (1.36–1.50)
Bipolar disorder	No	2,038,214 (99.94%)	203,782 (99.92%)	1 (ref)
	Yes	165 (0.08%)	1252 (0.06%)	1.32 (1.12–1.55)
Migraine	No	197,068 (96.63%)	1,990,109 (97.58%)	1 (ref)
	Yes	6879 (3.37%)	49,357 (2.42%)	1.43 (1.39–1.46)

OR: Odds ratio; 95%CI: 95% confidence interval; ref: reference category; PCP: Primary Care Physician; TIA: Transient Ischaemic Attack; HIV: Human Immunodeficiency Virus; AIDS: Adquired Immune Deficiency Syndrome.

**Table 4:** Previous medication use between cases and controls

Previous Medications	Categories	Cases (*n* (%)) *N* = 203,947	Controls (*n* (%)) *N* = 2,039,466	Crude OR (95% CI)
Corticoids for systemic use (Glucocorticoids)	Non use	1,756,946 (86.15%)	162,552 (79.7%)	1 (ref)
	0–30 days	23,529 (1.15%)	3688 (1.81%)	1.76 (1.69–1.82)
	31–365	97,827 (4.8%)	14,787 (7.25%)	1.7 (1.67–1.73)
	365+	161,164 (7.9%)	22,920 (11.24%)	1.6 (1.57–1.62)
Antivirals for systemic use	Non use	2,025,268 (99.3%)	200,749 (98.43%)	1 (ref)
	0–30 days	723 (0.04%)	291 (0.14%)	4.09 (3.57–4.68)
	31–365	3843 (0.19%)	888 (0.44%)	2.35 (2.18–2.53)
	365+	9632 (0.47%)	2019 (0.99%)	2.14 (2.04–2.25)
Immunosuppressants	Non use	2,035,287 (99.8%)	203,157 (99.61%)	1 (ref)
	0–30 days	2265 (0.11%)	490 (0.24%)	2.17 (1.97–2.39)
	31–365	719 (0.04%)	134 (0.07%)	1.87 (1.56–2.25)
	365+	1195 (0.06%)	166 (0.08%)	1.4 (1.19–1.64)
Antibacterials for systemic use	Non use	777,879 (38.14%)	45,965 (22.54%)	1 (ref)
	0–30 days	136,679 (6.7%)	25,353 (12.43%)	3.37 (3.31–3.43)
	31–365	541,039 (26.53%)	75,211 (36.88%)	2.52 (2.48–2.55)
	365+	583,869 (28.63%)	57,418 (28.15%)	1.75 (1.73–1.78)
Anxiolytics	Non use	1,669,397 (81.85%)	153,836 (75.43%)	1 (ref)
	0–30 days	76,617 (3.76%)	11,381 (5.58%)	1.84 (1.80–1.88)
	31–365	103,121 (5.06%)	17,975 (7.34%)	1.70 (1.67–1.73)
	365+	190,331 (9.33%)	23755 (11.65%)	1.45 (1.43–1.48)
Antipsychotics	Non use	1,950,135 (95.62%)	191,695 (93.99%)	1 (ref)
	0–30 days	11,729 (0.58%)	1267 (0.62%)	1.13 (1.07–1.20)
	31–365	21,457 (1.05%)	3094 (1.52%)	1.51 (1.46–1.57)
	365+	56,145 (2.75%)	7891 (3.87%)	1.48 (1.44–1.52)
Antiepileptic drugs (other than valproic acid)	Non use	2,012,359 (98.67%)	199,751 (97.94%)	1 (ref)
	0–30 days	8786 (0.43%)	1307 (0.64%)	1.52 (1.44–1.62)
	31–365	7572 (0.37%)	1243 (0.61%)	1.68 (1.58–1.79)
	365+	10,749 (0.53%)	1646 (0.81%)	1.57 (1.49–1.66)

OR: Odds ratio; 95%CI: 95% confidence interval; ref: reference category.

**Table 5:** Use of valproic acid and risk of clinical infections by herpesviruses.

	CASES *n* = 203,947	CONTROLS *n* = 2,039,466	Non-Adjusted OR † (95%CI)	Adjusted OR ‡ (95%CI)
**Exposure**				
Non use	203,644 (99.9%)	2,037,218 (99.9%)	1 (ref)	1 (ref)
Current use (0–30 days)	148 (0.1%)	1076 (0.1%)	1.38 (1.16–1.63)	0.84 (0.70–1.00)
Recent use (31–365 days)	65 (0.0%)	540 (0.0%)	1.20 (0.93–1.55)	0.79 (0.60–1.02)
Past use (>365 days)	90 (0.0%)	632 (0.0%)	1.42 (1.14–1.78)	0.94 (0.75–1.18)
Continuous duration *				
Non use	203,644 (99.9%)	2,037,218 (99.9%)	1 (ref)	1 (ref)
<91 days	52 (35.1%)	331 (30.8%)	1.57 (1.17–2.10)	1.02 (0.75–1.37)
91–365 days	57 (38.5%)	415 (38.6%)	1.37 (1.04–1.81)	0.83 (0.63–1.11)
>365 days	39 (26.4%)	330 (30.7%)	1.18 (0.85–1.64)	0.68 (0.48–0.95)

Exposure includes drugs containing valproic acid or related substances (sodium valproate or valpromide); OR: Odds ratio; 95%CI: 95% confidence interval; ref: reference category; †. crude model matched by age, sex, and month/year of the event; ‡. model adjusted by all variables included in Table 3; Table 4; *. Among current users.

**Table 6:** Use of valproic acid and risk of clinical infections by different herpesviruses.

	HHV-1/HHV-2 OR (95% CI)	HHV-3 OR (95% CI)	HHV-4/HHV-5 OR (95%CI)
**Exposure**			
Non use	1 (ref)	1 (ref)	1 (ref)
Current use (0–30 days)	0.89 (0.70–1.15)	0.92 (0.68–1.24)	0.97 (0.51–1.83)
Recent use (31–365 days)	0.87 (0.61–1.24)	0.80 (0.52–1.24)	0.84 (0.29–2.45)
Past use (>365 days)	1.04 (0.79–1.37)	0.88 (0.54–1.43)	0.79 (0.34–1.81)
**Continuous duration†**			
Non use	1 (ref)	1 (ref)	1 (ref)
<91 days	1.16 (0.77–1.74)	0.92 (0.56–1.52)	1.40 (0.49–4.02)
91–365 days	0.85 (0.56–1.30)	0.87 (0.56–1.36)	1.87 (0.77–4.53)
>365 days	0.71 (0.45–1.12)	0.95 (0.55–1.65)	0.15 (0.02–1.16)

Exposure includes drugs containing valproic acid or related substances (sodium valproate or valpromide). OR: Odds ratio; 95%CI: 95% confidence interval; HHV-1/HHV-2 (causing orolabial/genital herpes); HHV-3 (varicella/herpes zoster); HHV-4/HHV-5 (infectious mononucleosis). Results for HHV-6 (roseola infantum) and HHV-8 (Kaposi’s sarcoma) are not provided separately because models did not converge. Results of all herpes viruses combined are displayed in Table 5. †. among current users. ref: reference category.

## Appendix A. Procedures to Build the ICPC-BIFAP Medical Terms Dictionaries

Currently, two coding systems, with different levels of granularity, coexist in BIFAP: the International Classification of Primary Care (ICPC) and the International Classification of Diseases (ICD-9). The ICPC is the coding system for eight out of nine participant Autonomous Regions and its granularity is limited as compared with ICD-9 (686 vs. 23,222 codes, respectively).

To identify the episode of interest by the primary care physician (PCP), the EHR software contains an internal thesaurus, where a list of descriptors of diseases, signs, or symptoms is linked to the different dictionary codes. Often, these descriptors provide more detailed information than that in the corresponding code. Likewise, only for ICPC-based EHR software, new descriptors can be included at the local level, and the PCPs can also modify or add information to the selected episode descriptor. This EHR software flexibility results in a huge number of different descriptors in the BIFAP database (3.4 million).

To standardize this, BIFAP has developed its own research dictionary (ICPC-BIFAP) by adding, to the most frequently used descriptors, a fourth digit to the original three-digit ICPC code, increasing its granularity. In 2014, the ICPC-BIFAP dictionary included 5799 indexed terms. ICPC-BIFAP and ICD-9 codes covers about 93.2% of all diagnoses registered in BIFAP (116.7 million).

Concerning the autonomous region coding with ICD-9, given the big granularity of the medical terms dictionary, information received in BIFAP is already normalized and no further actions are needed.

To ease the management of the events for pharmacoepidemiological studies, specific case-finding algorithms (CFA) have been developed for an increasing number of outcomes. CFAs include related codes in both ICD-9 and ICPC-BIFAP dictionaries and might also include laboratory test results, additional health data, or text mining strategies. Text mining strategies are usually included to build CFAs given that, as commented previously, 6.8% of diagnoses in BIFAP are not mapped to any of the ICPC-BIFAP codes available.

## Appendix B. BIFAP Case-Finding Strategies to Identify Diseases Caused by Herpesvirus Infections

**Table A1:** International Classification of Diseases (ICD)-9 codes for diseases caused by herpesviruses.

ICD-9 Code	Description
052	Chickenpox
052.0	Postvaricella encephalitis
052.1	Varicella (hemorrhagic) pneumonitis
052.2	Postvaricella myelitis
052.7	Chickenpox with other specified complications
052.8	Chickenpox with unspecified complication
052.9	Varicella without mention of complication
054	Herpex simplex
054.0	Eczema herpeticum
054.1	Genital herpes
054.10	Genital herpes, unspecified
054.11	Herpetic vulvovaginitis
054.12	Herpetic ulceration of vulva
054.13	Herpetic infection of penis
054.19	Other genital herpes
054.2	Herpetic gingivostomatitis
054.3	Herpetic meningoencephalitis
054.4	Herpex simplex with ophthalmic complications
054.40	Herpes simplex with unspecified ophthalmic complication
054.41	Herpes simplex dermatitis of eyelid
054.42	Dendritic keratitis
054.43	Herpes simplex disciform keratitis
054.44	Herpes simplex iridocyclitis
054.49	Herpes simplex with other ophthalmic complications
054.5	Herpetic septicemia
054.6	Herpetic whitlow
054.7	Herpex simplex with other specified complications
054.71	Visceral herpes simplex
054.72	Herpes simplex meningitis
054.73	Herpes simplex otitis externa
054.74	Herpes simplex myelitis
054.79	Herpes simplex with other specified complications
054.8	Herpes simplex with unspecified complication
054.9	Herpes simplex without mention of complication
075	Infectious mononucleosis
078.5	Cytomegaloviral disease
771.1	Congenital cytomegalovirus infection
058.1	Roseola infantum
058.10	Roseola infantum, unspecified
058.11	Roseola infantum due to human herpesvirus 6
058.12	Roseola infantum due to human herpesvirus 7
058.21	Human herpesvirus 6 encephalitis
058.81	Human herpesvirus 6 infection
058.82	Human herpesvirus 7 infection

**Table A2:** International Classification of Primary Care (ICPC)-Base de datos para la Investigación Farmacoepidemiológica en Atención Primaria (BIFAP) codes for diseases caused by herpesviruses.

ICPC Code	BIFAP Subcode	Description
A77	9	Cytomegalovirus infection
A75	1	Infectious mononucleosis
A75	2	Epstein–Barr virus infection
A72	1	Chickenpox
D83	19	Herpetic stomatitis
D83	20	Herpetic gingivostomatitis
D83	26	Herpetic gingivitis
F16	10	Herpes simplex eyelid
F73	18	Herpes simplex eye
S71	1	Herpex simplex mouth/lip
S71	2	Herpetic whitlow
S71	3	Herpetic infection skin
S71	5	Herpes simplex
X90	1	Herpetic infection genital female
Y72	1	Herpetic infection genital male
Y72	999	Herpetic infection genital & annus male
W71	6	Herpes pregnancy
F73	20	Herpes keratitis
S71	4	Herpes not specified

**Table A3:** Text mining search strategies.

Specific Strings (in Spanish)
HERPES OR HERPET% OR HERPEX OR CITOMEGALOVIRUS OR CMV OR MONONUCLEOSIS OR VARICELA * OR EPSTEIN OR ROSEOLA† OR KAPOSI

* Excluded if string text identifying vaccination are also included; † Excluded if string text identifying a syphilitic etiology are also included.
